# Depressive Disorder With Panic Attacks After Replacement of an Intrauterine Device Containing Levonorgestrel: A Case Report

**DOI:** 10.3389/fpsyt.2020.561685

**Published:** 2020-08-28

**Authors:** René Zeiss, Carlos Schönfeldt-Lecuona, Maximilian Gahr, Heiko Graf

**Affiliations:** Department of Psychiatry and Psychotherapy III, Ulm University Hospital, Ulm, Germany

**Keywords:** intrauterine device, levonorgestrel, depression, anxiety, case report

## Abstract

The levonorgestrel-releasing intrauterine system (LNG-IUS) is used as hormonal contraception by millions of women worldwide. It is considered as a safe device with low rates of systemic adverse drug reactions (ADRs). However, an emerging evidence suggest mood changes as ADRs. Whereas most of these studies report psychiatric ADRs after the first implantation of the LNG-IUS, it has to be considered that these may also occur after replacement, even when psychiatric symptoms were not evident at the time of the initial insertion. A potential explanation for the development of psychiatric ADRs in subsequent LNG-IUS may rely on fluctuations of sex hormones throughout the female life cycle with changing windows of vulnerabilities for developing mood disorders. Thus, the reliable contraception for women remains a continual challenge. We present the case of a 41-year-old woman that used the LNG-IUS (Mirena^®^) for contraception over 5 years without any complaints. Within the first weeks after insertion of the second LNG-IUS, she developed a depressive syndrome and anxieties. An extensive somatic, including gynecological examination revealed no pathological findings and a mental disorder was suggested. Due to the patient´s request and the recommendation of her psychiatrist, the device was removed and led to a remission of her mental complaints up to a 6- and 12-months follow-up. Beyond the mood changes considerably affecting her quality of life, the patient raised the concerns that she has never been informed about potential ADRs on mental health and her remarks regarding the potential association between psychiatric symptoms and the LNG-IUS were considered as groundless. With this case, we strengthen previous observations regarding mood changes under LNG-IUS. Moreover, we illustrate that psychiatric symptoms may also occur as ADRs during the subsequent insertion. Thus, we emphasize that psychiatric symptoms have to be clearly communicated as ADRs to patients with LNG-IUS within a written informed consent and should be routinely examined by gynecologists.

## Background

The levonorgestrel‐releasing intrauterine delivery system (LNG-IUS) is registered in more than 120 countries worldwide and considered as an effective and safe device for contraception for up to 5 years, and for the treatment of several gynecological conditions, e.g. menorrhagia [5]. The most frequent adverse drug reactions (ADRs) of LNG-IUS are thought to be menstrual irregularities and changes in bleeding patterns (1). In contrast to oral contraceptives, systemic effects of LNG-IUS and drug-drug interactions have been underestimated in clinical practice for years. Due to the localized release of the drug, blood serum levels of levonorgestrel are lower compared to other hormonal contraceptives, and ADRs were supposed to occur less pronounced or mainly in the initial months of use. However, an increased risk of breast cancer (2) and an emerging evidence regarding mood disorders and suicide attempts (3–6) arises the question of clinically relevant systemic and in particular psychotropic effects of LNG-IUS. Previous studies investigated ADRs only over a time period of 3 to 5 years after implantation (7–9). Studies on removal and insertion procedures of the second LNG-IUS mainly focused on bleeding patterns (10, 11). Considering the pharmacokinetic profile of LNG-IUS with an increase in serum levels within the first weeks after implantation (12), an increase in ADRs including psychiatric symptoms after the replacement seem plausible, but corresponding reports are scarce (13).

We illustrate the case of a 41-year-old woman who developed a severe depressive syndrome with panic attacks and suicidal ideations after insertion of the second LNG-IUS after 5 years. These symptoms remitted after the removal of the device.

## Case Presentation

**Figure 1:**
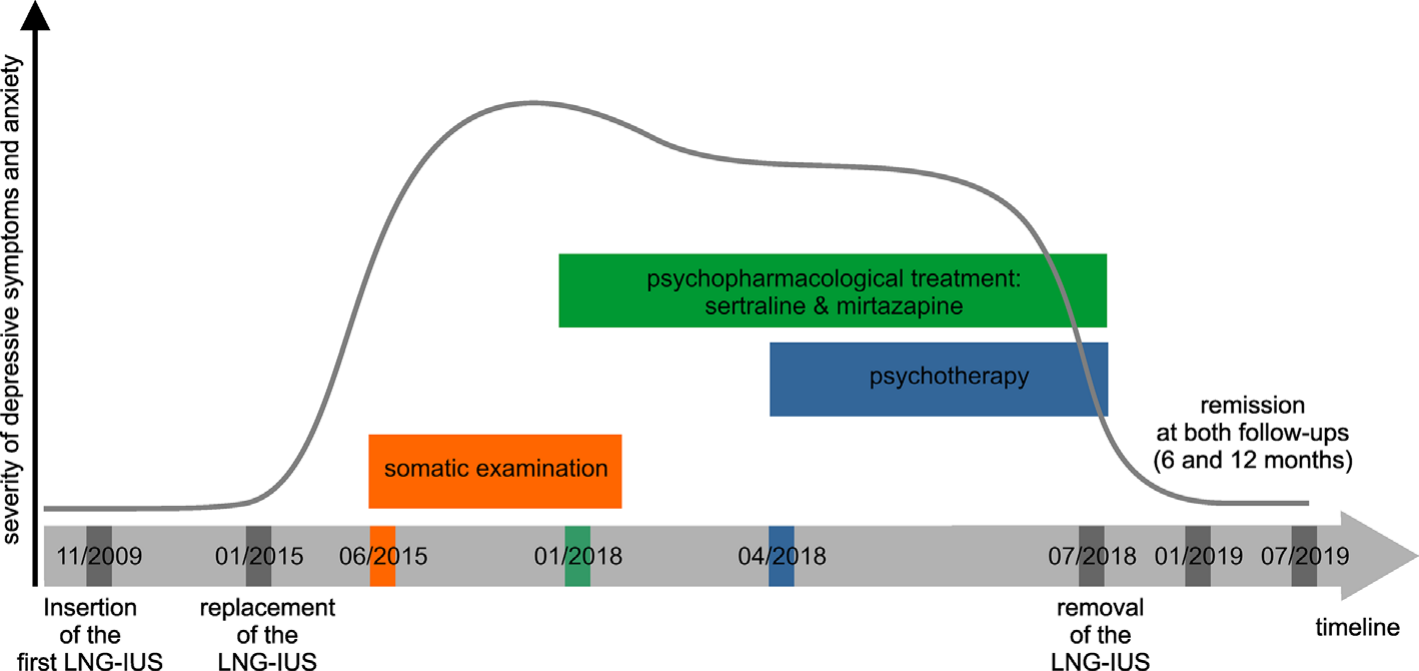
Timeline showcasing the course of psychiatric symptoms, the somatic examination, the psychopharmacological, psychotherapeutic treatment and the insertion, replacement and removal of the LNG-IUS over time. Somatic examinations included gynecological, cardiological and rheumatological investigations as mentioned in the main text. Psychotherapeutic treatment comprised five supportive consultations. LNG-IUS, levonorgestrel-releasing intrauterine system.

The 41-year-old housewife was married for 20 years. After the birth of her youngest child 8 years ago, an LNG-IUS (Mirena^®^) was chosen as a long-term contraception owing to the patients` preference. Of note, there was no other gynecological indication (e.g. menorrhagia) for the insertion of the device. Except for daily tasks involving her household and taking care of her four children, no psychosocial stress factors were reported. There was no history of psychiatric disorders or general medical condition, neither in the past nor within her family. The patient reported from a regular menstrual cycle and denied current or previous pre- or perimenstrual syndromes. Moreover, there was no other medication than the LNG-IUS within the last few years. After 5 years of implantation of the first LNG-IUS and approximately 2.5 years from present, a second LNG-IUS was inserted. The removal of the first and the insertion of the new LNG-IUS occurred on the same day. Within the first weeks after the insertion of the second device, she experienced an increasing psychomotor restlessness that was pronounced in the evening and during the night. She also reported from episodes of tachycardia and experienced an irregular heartbeat. Within the next 6 months, symptoms worsened and she developed sleep disturbances, anxiety in the night, emotional lability and suicidal ideations. She also reported of somatic complaints such as shivering, hot flashes, and pelvic pain with variable intensity. Repeated medical examinations by the family doctor, including ECG, blood tests (thyroid hormones, glucose, infection parameters, liver and kidney values, electrolytes, differential blood count) were without any significant findings. A cardiologic examination including a long-term ECG, exercise ECG and cardiac ultrasound revealed supraventricular extrasystoles without any other pathological findings. These internal and cardiological non-findings could however, not calm the patients´ worries down and had no impact on her well-being. Her anxieties still worsened with an increased frequency of panic attacks including the fear to faint or even to die and led to an emergency call. Due to the pelvic pain, a rheumatological examination including the determination of autoantibodies (antinuclear antibodies, ANA; anti-neutrophil cytoplasmic antibodies, ANCA) was conducted but also without any pathological findings. Several gynecological examinations led to the presumptive diagnosis of a premenopausal syndrome in the context of an early menopause. However, since the previous somatic examinations revealed no pathological findings, a mental origin was assumed and a psychiatric treatment was recommended. At this time the duration of the symptomatic has worsened over the course of 3 years. **Figure 1** shows a timeline with information about the course of symptoms, the performed examinations and the treatment.

## Therapeutic Intervention

Due the suspected diagnosis of a depressive and anxiety disorder, the family doctor initiated an antidepressive medication with sertraline (100 mg OPD) and mirtazapine (7.5 mg OPD). This medication was taken daily for 6 months. In addition, lorazepam (0.5–1 mg) was prescribed as medication on demand. With medication, she reported from a minor improvement of sleep disturbances and from a remission of panic attacks, but still, higher levels of anxiety, depressed mood, inner restlessness, rumination and emotional lability persisted. Thus, the patient consulted a psychiatrist and underwent a supportive psychotherapy for five sessions in total. At this time, psychometric subjective measures of depressive mood conducted with the Beck depression inventory^1^ (BDI), revealed a sum-score of 42, indicating a severe depressive syndrome. After psychoeducation regarding depressive symptoms, the psychiatrist informed the patient about the potential association between changes in mood and the use of hormonal contraceptives. The psychiatrist also recommended the removal of the LNG-IUS. The gynecologist initially denied the potential association between the psychiatric symptoms and the LNG-IUS since changes in mood or anxiety were not evident during the first period of the LNG-IUS contraception. Nevertheless, the LNG-IUS was removed due to patient’s request.

## Outcome and Follow-Up

Two weeks after removal of the LNG-IUS, psychiatric and in particular depressive symptoms improved markedly up to a BDI sum-score of 18, indicating a mild depressive syndrome, despite reduction of antidepressants. After two further weeks, the antidepressive medication was stopped. Four weeks after removal of the LNG-IUS, the patient reported that depressive symptoms and anxiety remitted completely (BDI sum-score: 6). In a 6- and 12-months follow-up, there were no clinically relevant symptoms of depression or anxiety (BDI: 2 respectively 2) observed.

## Discussion

In the present case, we demonstrate a patient that developed depressive symptoms and anxiety after the insertion of the second LNG-IUS. After the removal of the device, psychiatric symptoms remitted completely. We are aware that we observed a temporal association that may not proof the causal relationship between the replacement of the LNG-IUS and the depressive syndrome. Though, considering the criteria of the World Health Organisation (WHO) drug-monitoring-center (15), the definitions of Edwards and Aronson (16), and the Naranjo algorithm (17), a causative role of the LNG-IUS for developing these psychiatric symptoms has to be classified as “possible” or even as “probable/likely”. A re-challenge with Mirena^®^ was not conducted for ethical reasons. Despite the well-known discussion regarding the latency of antidepressive medication in which they achieve their full effects (18), one may also assume that the clinical improvement of depressive symptoms in our case was due to the antidepressive medication or the supportive psychotherapeutic assistance. Although we cannot fully exclude the effects of medication or psychotherapy, it is of note, that this argument is not eligible to attenuate our observation of developing psychiatric symptoms shortly after the insertion of the second LNG-IUS. Nevertheless, we are aware that we were not able to pinpoint the exact cause for depression and anxiety. Although the removal of the first and the insertion of the second LNG-IUS occurred on the same day, we also cannot fully exclude that the removal of the first device mainly contributed to the development of psychiatric symptoms due to hormonal changes rather than the insertion of the second device.

The recent literature support a good benefit-risk assessment for LNG-IUS as contraception with low systemic effects (19, 20), presumably due to the local application and local release of LNG into the cavum uteri. The use of LNG-IUS is even recommended for contraception by professional societies including the American Academy of Pediatrics (AAP) and the American College of Obstetricians and Gynecologists (ACOG) in adolescents (21, 22).

In contrast to this recommendation, a nationwide prospective cohort study in Denmark observed an increase in suicide attempts and even suicide associated to the use of LNG-IUS (3). Notably, the highest relative risk of suicide attempt was found for adolescent women and increased twofold one month after insertion of the LNG-IUS compared to those who had never used hormonal contraception (3). In accordance with these findings, a recent Danish national register study reviewed the records of over one million women and observed that the use of LNG-IUS was associated with subsequent antidepressant use and first diagnosis of depression (4). In addition, a cohort study investigating psychiatric ADRs associated with LNG-IUS in the UK general practice, found significant associations between levonorgestrel exposure and records of anxiety and sleep disturbances (6).

Thus, our observation is in line with previous studies reporting a burden of affective symptoms in LNG-IUS users, suggesting a 34-percent higher risk of depression in women using the LNG-IUS (4). The underlying potential mechanism may rely on a centrally-mediated sensitization of autonomic and hypothalamic-pituitary-adrenal-axis responsivity with increased heart rates and higher cortisol levels, even when compared to oral contraceptives, the copper spiral or a natural cycle (5). Of note, women choosing the LNG-IUS are mostly unaware of the potential for the development of mood systems and these ADRs are rarely communicated routinely by doctors. Moreover, women are believed that their fears about such effects were dismissed as groundless (23).

Whereas most of these studies investigated mood effects after the first insertion of the device, it is of note that our patient developed symptoms of depression and anxiety after the implantation of the second LNG-IUS. In general, it is well known that side effects of medication occur even after the intake of years. However, considering the increasing evidence that in particular women are at higher risk to develop mood symptoms due to significant fluctuations of sex hormones over time (24), one may argue that the implantation of LNG-IUS in such potential windows of vulnerability, e.g. pre-/peri- or postmenopausal periods, may considerably increase the risk of psychiatric ADRs. Alternatively, it is of note that locally released LNG by IUSs lead to endometrial concentrations that are 200-800 times those found after daily oral use (25). Considering the endometrial thinning after the prolonged use of LNG-IUS as in our case, one may assume an altered pharmacokinetic profile of LNG-IUS within the first weeks after the insertion of the second device, accompanied with higher risks of systemic effects. However, this remains speculative and the pharmacokinetic alterations and in particular, corresponding systemic or psychiatric effects after the replacement of the LNG-IUS await an empirical investigation in future studies.

Nevertheless, our case is supposed to strengthen the awareness of every health worker for potential ADRs, and in particular psychiatric symptoms. While it is quite usual to control the positioning of the LNG-IUS *via* ultrasound to ensure adequate contraception, it should be equally usual for gynecologists to examine the patient’s mental condition, not only after first insertion of an LNG-IUS and over time, but also in particular after replacement and insertion of the subsequent device. Moreover, this case should emphasize the inevitable necessity to clarify potential psychiatric side effects in terms of a written informed consent.

## Patient’s Perspective

Since I was very satisfied with the LNG-IUS, I decided to replace the device after the recommended 5 years. Shortly after the insertion of the second LNG-IUS, I became increasingly nervous, felt an inner unrest and during the following weeks, I felt sad and had even suicidal thoughts. I consulted several medical doctors, but they found no physical cause for my complaints. My family doctor told me that I might have a depressive disorder, prescribed antidepressants and referred me to a psychiatrist. After the intake of the antidepressants, I felt somewhat relieved as the intensity of the symptoms became less, but nevertheless, I still felt sick and limited in various settings of my daily life and my quality of life heavily decreased. My psychiatrist told me that the LNG-IUS might be causative for my mental symptoms and recommended to remove it. In contrast, my gynecologist mentioned that the LNG-IUS may not have an impact on my mental status. Since may mood still further decreased and since I previously suggested an association between the LNG-IUS and my psychological complaints by myself, I repeatedly asked my gynecologist to remove the device. Within the next few weeks after the removal and although I quit antidepressive medication, I felt increasingly better, my mood was getting better and my inner restlessness remitted. I have no doubts that my mental complaints were associated to the LNG-IUS considering the strong association between the onset of my symptoms and the removal. I am aware that every medication has side effects that cannot be predicted. Nevertheless, I am still disappointed and angry since my concerns were not taken seriously and more important, I was not informed regarding potential mental symptoms before the insertion of the device, neither by verbal information, nor by a written product information.

## Data Availability Statement

The original contributions presented in the study are included in the article/supplementary material; further inquiries can be directed to the corresponding author.

## Ethics Statement

Written informed consent was obtained from the individual(s) for the publication of any potentially identifiable images or data included in this article.

## Author Contributions

RZ, CS-L, and HG wrote the manuscript with support from MG. CS-L was in contact with the patient. All authors contributed to the article and approved the submitted version.

## Conflict of Interest

The authors declare that the research was conducted in the absence of any commercial or financial relationships that could be construed as a potential conflict of interest.

## Footnotes

1 0–13: minimal depression; 14–19: mild depression; 20–28: moderate depression; 29–63: severe depression (14).
